# Supplementary figures and images for: Migration of Myogenic Cells Is Highly Influenced by Cytoskeletal Septin7
Source: Cells. 2023 Jul 11;12(14):1825. doi: 10.3390/cells12141825 (PMC10378681; doi:10.3390/cells12141825)

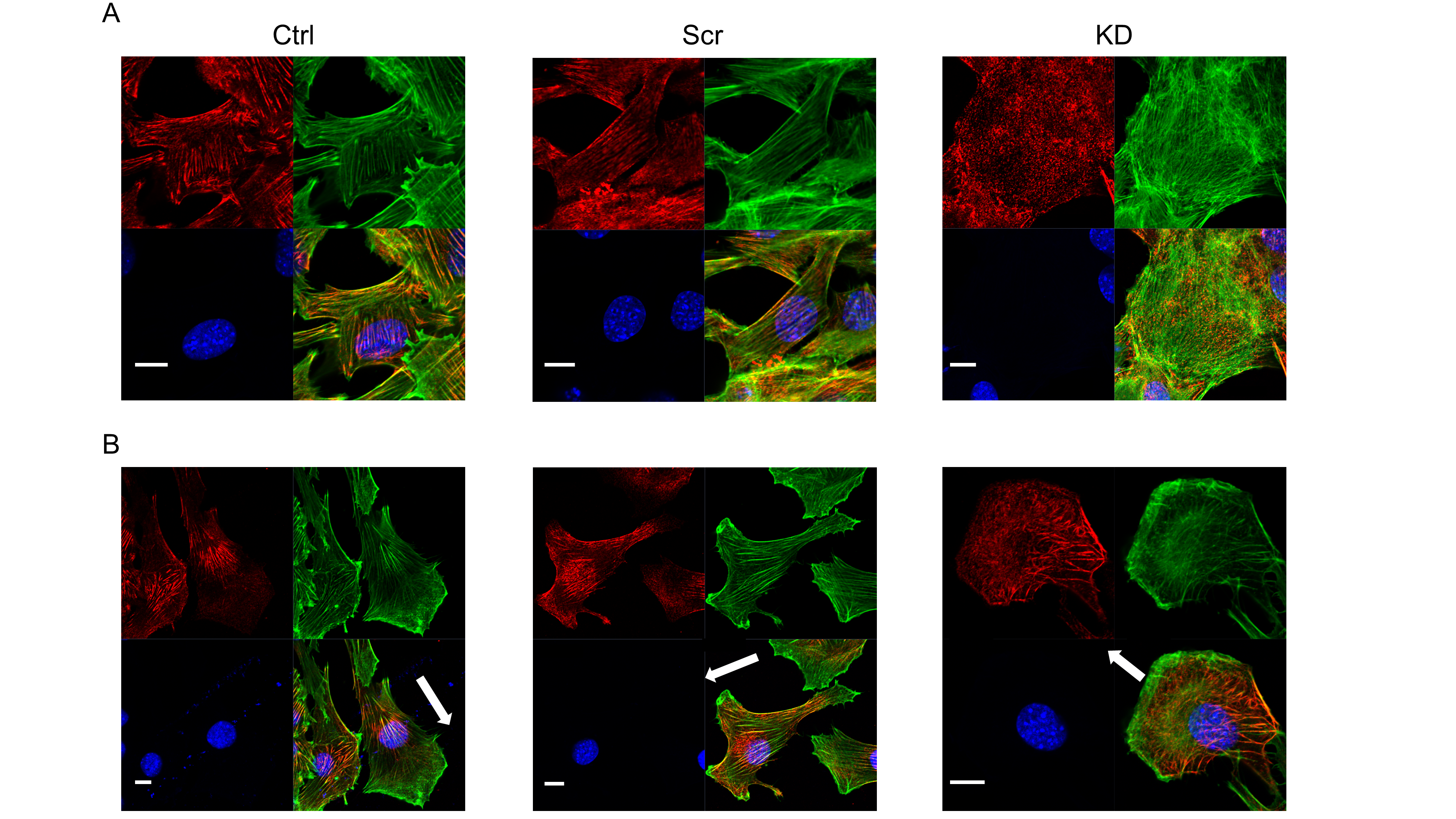

Supplement: Supplementary file 1 [file cells-12-01825-s001.zip › Figure S1.tif]

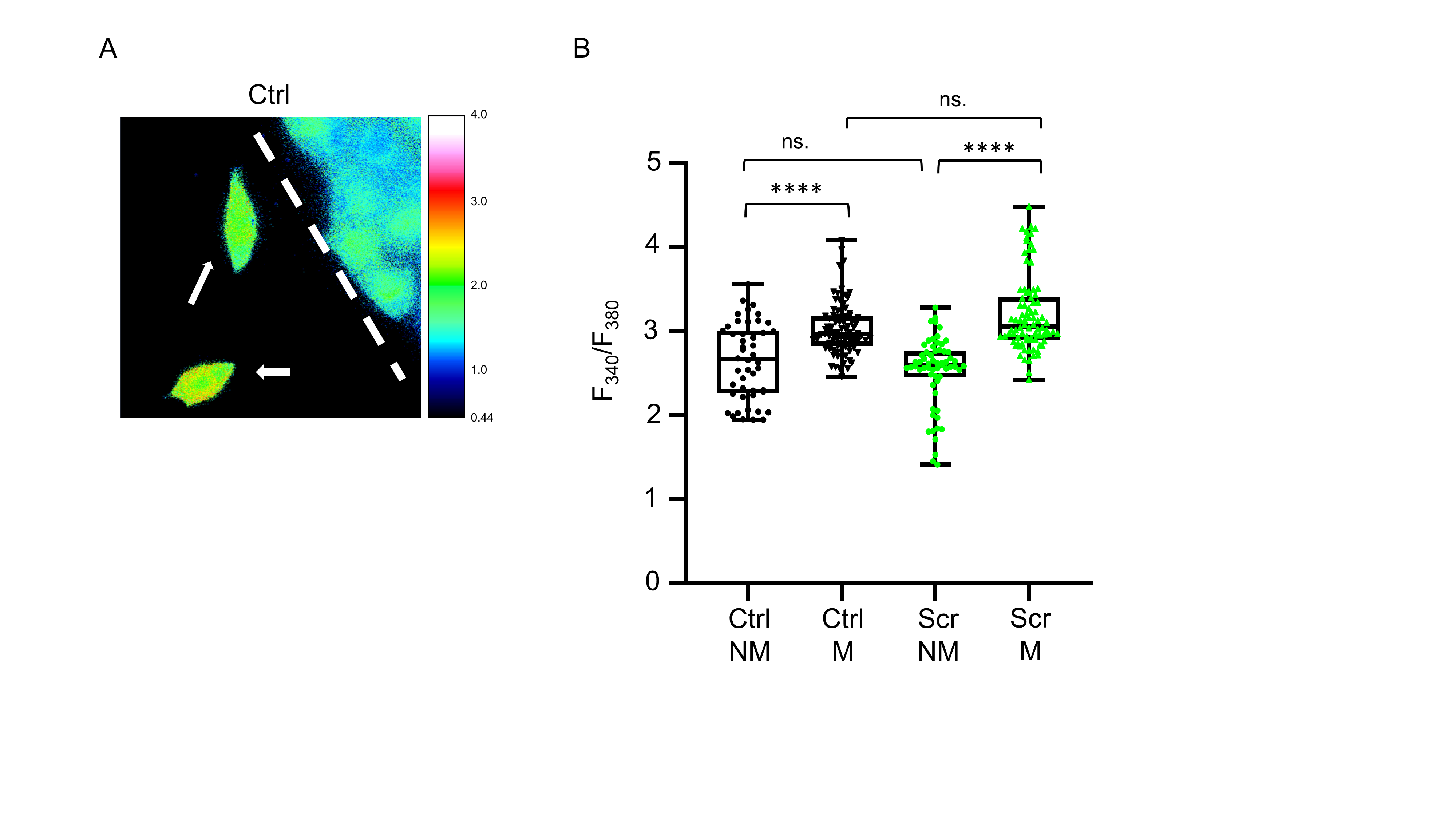

Supplement: Supplementary file 1 [file cells-12-01825-s001.zip › Figure S2.tif]

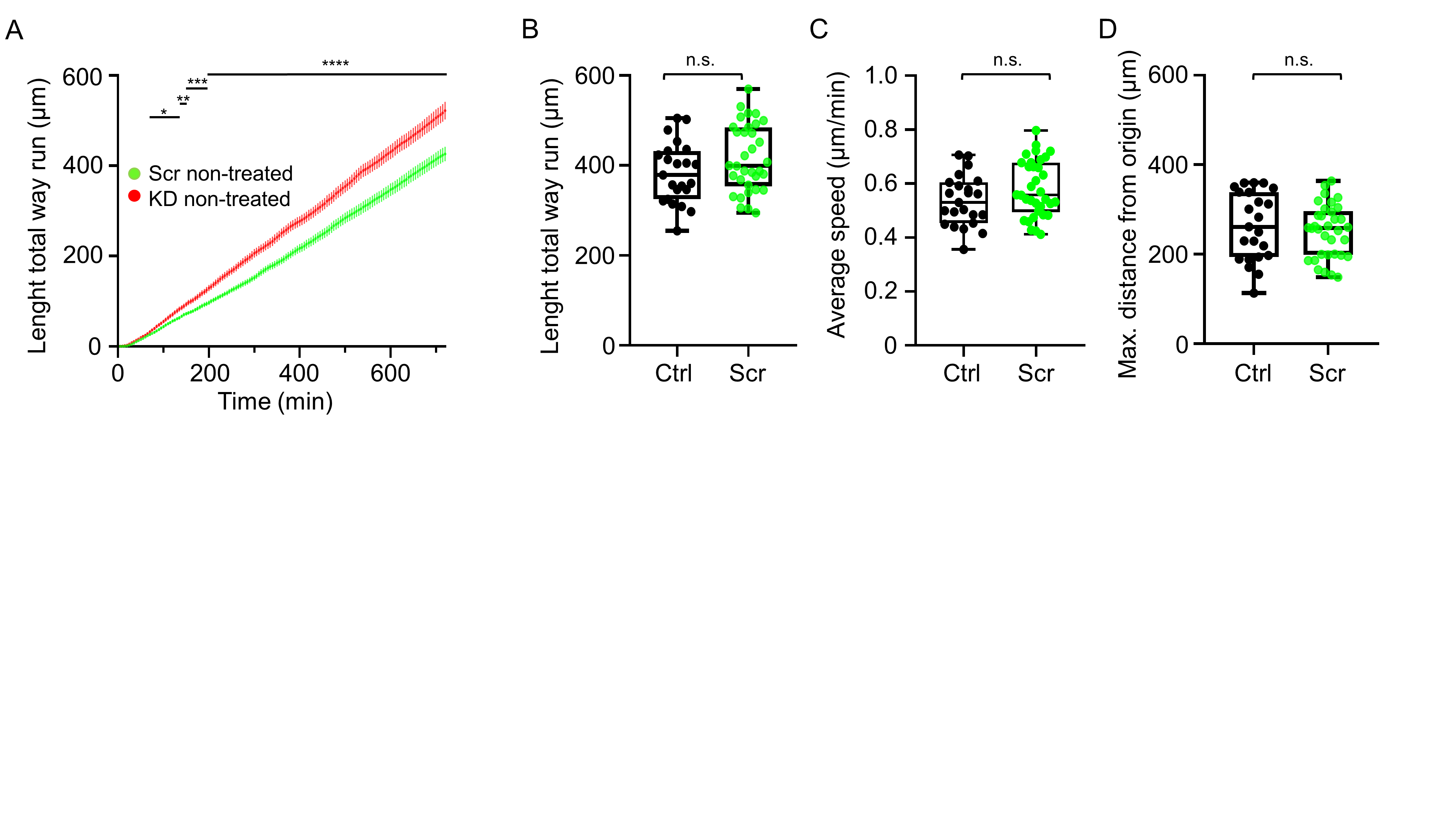

Supplement: Supplementary file 1 [file cells-12-01825-s001.zip › Figure S3.tif]

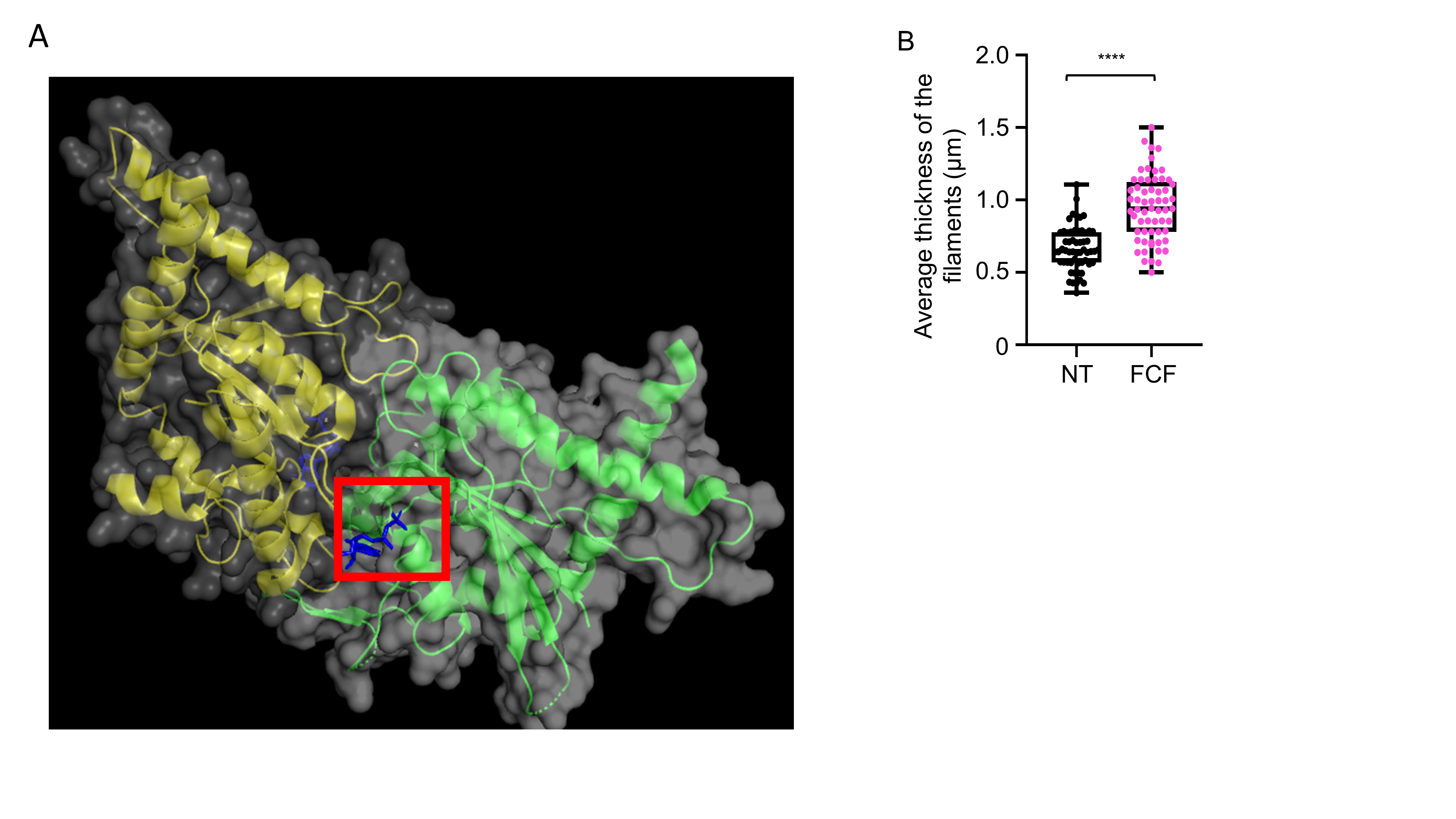

Supplement: Supplementary file 1 [file cells-12-01825-s001.zip › Figure S4.tif]

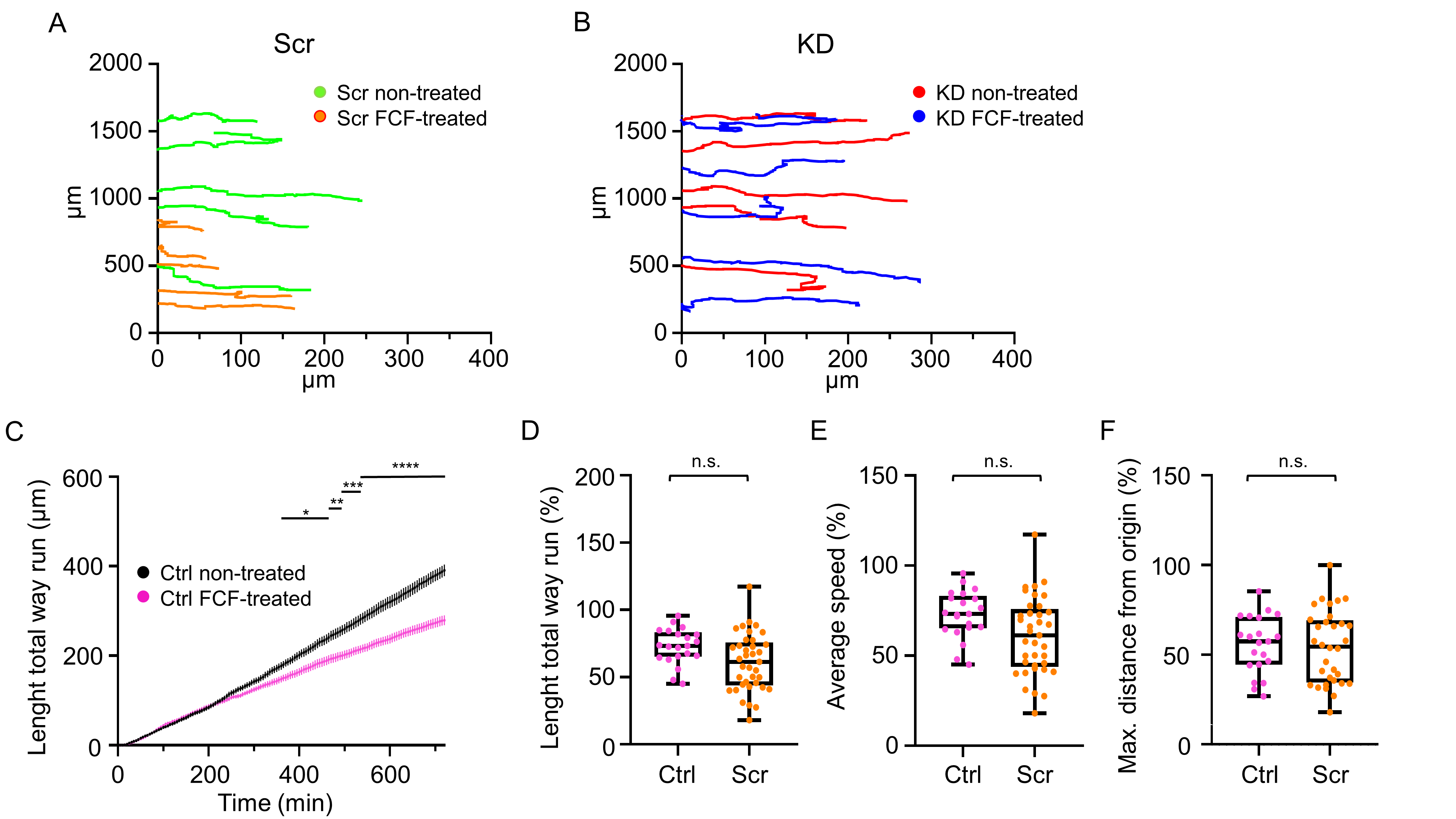

Supplement: Supplementary file 1 [file cells-12-01825-s001.zip › Figure S5.tif]
